# Radiomic features of CECT and SUVmax of dual-tracer PET/CT reveal PD-L1 spatial heterogeneity in PDAC

**DOI:** 10.1186/s40644-025-00960-3

**Published:** 2026-02-14

**Authors:** Sashikanta Swain, Sudipta Mohakud, Srijani Mandal, Siba Narayan Padhi, Bramhadatta Pattnaik, Sunita Gupta, Sipra Rout, Pravash Ranjan Mishra, Kanhaiyalal Agrawal

**Affiliations:** 1 Department of Anatomy, All India Institute of Medical Sciences, Bhubaneswar, India; 2 Department of Radiodiagnosis, All India Institute of Medical Sciences, Bhubaneswar, India; 3 Department of Nuclear Medicine, All India Institute of Medical Sciences, Bhubaneswar, India; 4 Department of Surgical Gastroenterology, All India Institute of Medical Sciences, Bhubaneswar, India

## Abstract

Pancreatic ductal adenocarcinoma (PDAC) is characterized by limited responsiveness to immunotherapy due to spatial heterogeneity of PD-L1 expression. Invasive biopsy-based assessments may fail to capture this heterogeneity. This study aims to evaluate non-invasive imaging biomarkers to predict the expression of PD-L1 in 52 PDAC patients using CECT and F-18 FDG PET/CT and Ga-68 FAPI-04 PET/CT imaging. Intensity histogram and Gray Level Co-occurrence Matrix (GLCM) features were extracted from CECT using IBEX software. PD-L1 expression was validated by immunohistochemistry, RT-PCR, and Western blotting. Correlation between PD-L1 expression, SUVmax of F-18 FDG, and FAPI-04 PET/CT and radiomic CECT features were assessed. PDAC tissues demonstrated multimodal intensity histograms with lower histogram peak height correlated with high PD-L1 expression. A moderate positive correlation was observed between PD-L1 expression and FDG uptake (R^2^ = 0.36) and a weaker correlation with FAPI uptake (R^2^ = 0.19). This Multi-parametric imaging can enhance immunohistochemistry, aiding in patient stratification for immunotherapy and overcoming the limitations of single-site biopsies.

**Supplementary Information:**

The online version contains supplementary material available at 10.1186/s40644-025-00960-3.

## Introduction

Pancreatic ductal adenocarcinoma (PDAC) is the fourth leading cause of cancer-related deaths worldwide [1]. Its incidence has been steadily rising in recent years, primarily due to the absence of effective early detection methods [2–4]. The poor prognosis of PDAC’s can be linked to late-stage diagnosis, therapy failure, and intrinsic chemoresistance [5–8]. Although immunotherapy has effectively treated many other cancers, it has not demonstrated meaningful clinical benefits in PDAC [9].

Tumor cell PD-L1(Programmed Death-Ligand 1) expression predicts immunotherapy response [10]. Unlike binary expression models, PD-L1 is often expressed in a heterogeneous pattern across tumor and stromal compartments, reflecting the dynamic interplay between tumor cells and the immune microenvironment [11]. This heterogeneity significantly impacts the response to immune checkpoint inhibitors (ICIs), particularly those targeting the PD-1/PD-L1 axis. An accurate assessment of PD-L1 expression is crucial for improving immunotherapy efficacy [12]. Sequencing and molecular profiling from biopsy samples provide information on PD-L1 dispersal, but the intratumor heterogeneity in biopsy questioned its efficacy.

In PDAC assessment, CECT is the first choice because of its widespread accessibility and non-invasive technique. CECT heterogeneity is complementary to PET-based semiquantitative parameters [13, 14]. According to recent research, the accumulation of fluorine-18 (F-18) 2-deoxy-2-fluoro-D-glucose (FDG) in PET is closely linked to the expression of PD-L1 within tumor cells [15]. With a high tumor uptake rate and minimal background noise, fibroblast activation protein inhibitor (FAPI) PET/CT recently targets fibroblast activation protein (FAP) and stromal fibrosis. FAP possesses both endopeptidase and dipeptidyl peptidase activities and belongs to the dipeptidyl peptidase four protein family [16]. Over 90% of epithelial carcinomas have stromal fibroblasts that have high levels of FAP expression [17]. According to the heterogeneity of CAFs (Cancer-Associated Fibroblasts) and their interaction with TME, CAFs promote PD-L1 expression. FAP is a marker expressed by a subset of CAFs, and CAFs have the ability to alter the extracellular matrix and control a number of biological processes linked to metastasis and tumor immunity [18–20].

Integrating imaging modalities (CECT and PET/CT) with molecular analyses will highlight PD-L1 expression across tumor grades. Specifically, areas demonstrating higher metabolic activity or altered stromal architecture.

## Materials & methodology

**Fig. 1:**
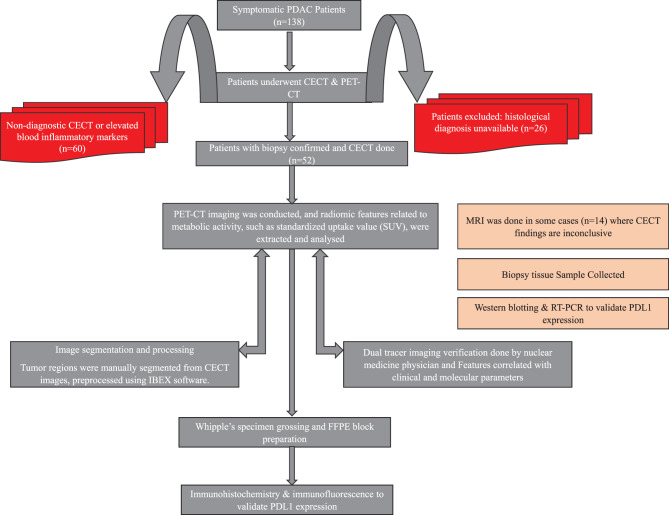
Study Workflow for patient inclusion, imaging, and Molecular analysis in PADC cohort. Flowchart depicting the selection and stratification of symptomatic pancreatic ductal adenocarcinoma(PDAC) patients (*n* = 138) undergoing contrast-enhanced computed tomography (CECT) and PET-CT imaging

**Fig. 2:**
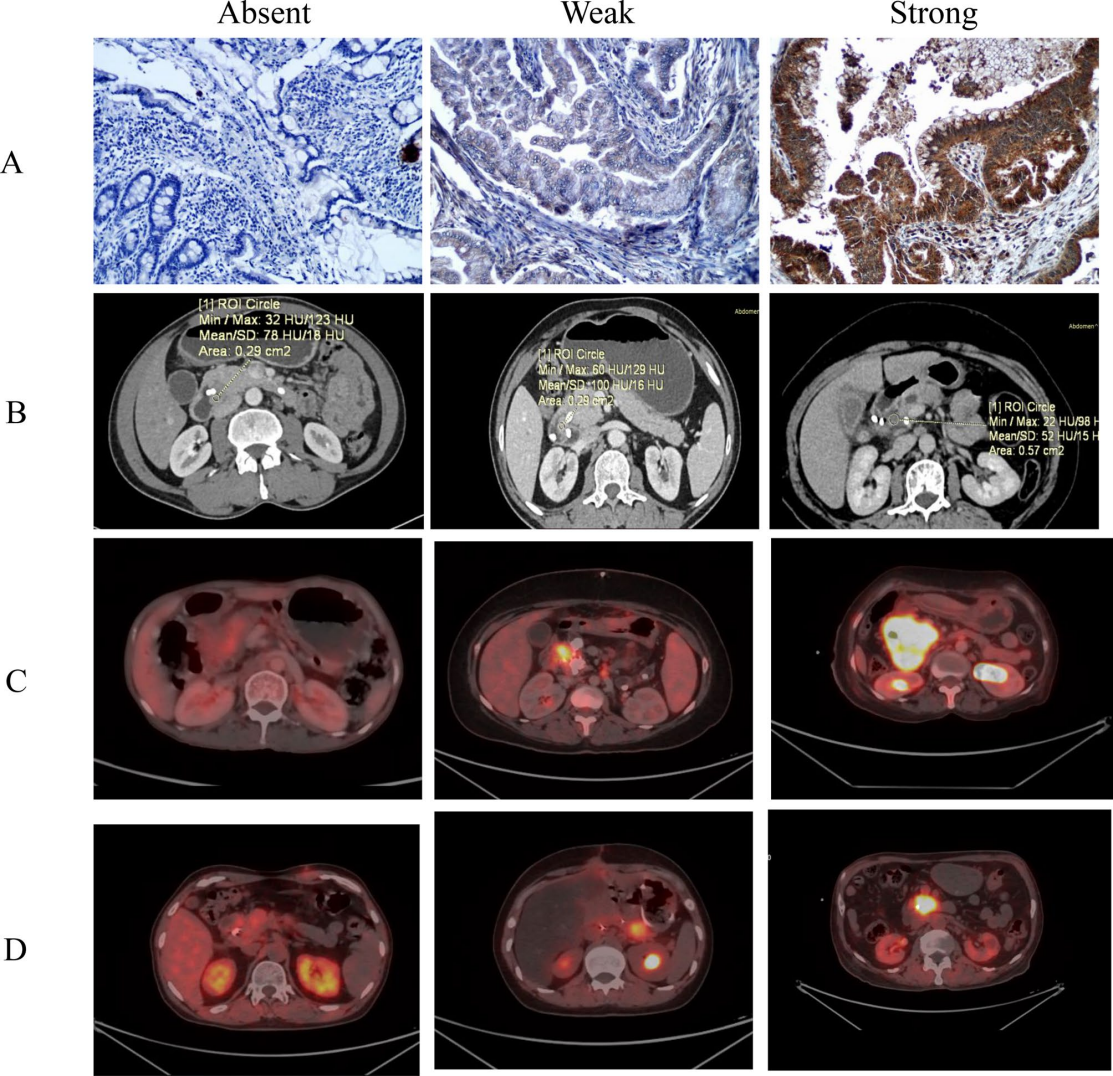
(**A**) Immunohistochemical staining showing variation in pd L1 expression in PDAC tissue, (**B**) CECT scans showing variation in PD L1 expression, (**C**) FDG PET CT showing variation in PD L1 expression, (**D**) FAPI PET CT showing variation in PD L1 expression

This is a prospective observational single-arm study. The Institute Ethics Committee approved the study (IEC/AIIMS BBSR/PhD Thesis/2023–24/04). Biopsy-proven, treatment-naive pancreatic cancer patients (*n* = 52) were included in the study. Patients with inflammatory blood reports were excluded. Patient demographic data and clinical history were noted (Table 1). Then, the patients underwent CECT, FDG, and FAPI PET/CT scans within one week of each scan modality (Fig. 1). Surgical samples are collected after the surgery for Immunohistochemistry (Fig. 2A).

**Table 1:** Clinicopathological characteristics of PDAC Patients (*n* = 52)

Variable		PDL1 Expression		
		Negative	Positive	*P* value
Gender	Female	8(15.4%)	13(25%)	0.4991
	Male	16(30.8%)	15(28.8%)	
Age(years)		62.3±6.4	68.3±5.7	0.418
CEA > 5 ng/ml		23(44.2%)	29(55.8%)	0.0821
T stage	T1/T2	16(30.8%)	17(32.7%)	0.8764
	T3/T4	8(15.4%)	11(21.2%)	
N stage	N0	15(28.85%)	12(23.08%)	0.0907
	N1/N2	9(17.31%)	16(30.77%)	
Grade	I	11(21.2%)	9(17.3%)	0.4680
	II	13(25%)	19(36.5%)	
FDG SUVmax > 7		14(26.9%)	25(48.1%)	0.0223
< 7		10(19.2%)	3(5.8%)	
FAPI SUVmax > 7		7(13.5%)	24(46.2%)	
< 7		17(32.7%)	4(7.7%)	< 0.0001

### CECT

Non-contrast CT (NCCT) was acquired first, followed by CECT imaging using a 256-slice multidetector CT scanner (SIEMENS SOMATOM Definition Flash, Germany). After intravenous injection of 80 mL of iodinated contrast agent at a rate of 3 mL/sec via an automated pressure injector, imaging was performed in two dynamic phases: The late arterial phase was acquired 20 seconds after contrast injection. The portal venous phase was acquired 40 seconds post-injection. A 20 mL normal saline flush was administered immediately after contrast injection at a 3 mL/sec rate to optimize vascular enhancement. Tumor localization and delineation were carried out in the portal venous phase by a radiologist with over 8 years of experience in abdominal imaging, ensuring consistency and diagnostic accuracy.

### Image segmentation and registration

Image segmentation and registration were performed using the IBEX (Imaging Biomarker Explorer) software to ensure accurate and reproducible radiomic analysis of CECT datasets. Manual segmentation of regions of interest (ROIs) was carried out on the portal venous phase of axial CECT images (Fig. 2(B)). Segmentation was performed slice-by-slice to account for three-dimensional tumor volume, capturing the lesion’s solid and heterogeneous components. Segmentation protocols followed an enhanced region that defined tumor area to ensure consistency across slices and reduce inter-observer variability. Adjacent vessels or necrotic areas were excluded when not part of the enhancing tumor, and the pancreatic parenchyma was carefully delineated in normal cases.

In cases where CECT images are inconclusive or affected by artifacts, MRI scans performed closest to the CECT date are considered for marking the tumor area in CECT.

### Evaluation of CECT imaging data

IBEX software was used to evaluate the CECT imaging data, and every slice (approximately 2500) was examined for each patient. In tumor regions of patients with PDAC, regions of interest (ROI) were particularly chosen. Intensity histograms were then plotted to represent the pixel intensity distributions within these tumor regions. Similarly, the ROIs in healthy pancreatic tissue in the same patients were chosen, and intensity histograms were produced. The evaluation of tumor-specific textural defects and alterations is made easier by directly comparing pixel intensity distribution patterns between malignant and normal pancreatic tissue.

### PET/CT

#### Patient preparation of PET/CT

Patients had no food for 4 hours before the FDG PET/CT. 0.1 mCi/kg body weight of FDG was administered intravenously. FDG PET/CT images were acquired 45–60 minutes after tracer injection (Fig. 2C). For Ga-68 FAPI PET-CT, radiotracer tagging was performed in the in-house radiopharmacy using a Ge-68/Ga-68 generator and FAPI cold kit, following the standard protocol. Ga-68 FAPI dose of 0.05–0.06 mCi/kg body weight was administered intravenously (Fig. 2D).

### Image acquisition

Whole body image acquisition was done from head to mid-thigh level after 45–60 minutes of injecting the radiopharmaceutical using a Discovery MI DR PET/CT scanner (GE Medical Systems, Milwaukee, WI). First contrast-enhanced CT scan images were acquired with a multidetector 128-slice spiral detector, followed by PET scanning. The images were reconstructed as transaxial, coronal, and sagittal images.

### Image analysis

Scans were read by a Nuclear Medicine physician with 13 years of experience. Any pathologically increased tracer uptake apart from known physiological tracer uptake was reported as abnormal, and SUVmax of the lesion was recorded in both studies.

### Data analysis

The scan was visually analyzed first. Increased tracer uptake in the primary tumor compared to the liver tracer uptake was considered positive for malignancy. To assess the ability of PET SUVmax to predict PD-L1 expression in pancreatic cancer, ROC curve analyses were performed for both FDG and FAPI PET-derived SUVmax values.

### PD L1 expression analysis

FFPE (Formalin-Fixed Paraffin-Embedded) blocks were derived from the primary tumors (*n* = 52). For comparison, normal pancreas tissues of 21 deceased persons with road traffic accidents were collected from the mortuary with written permission from the relatives. Subsequently, immunohistochemical and histopathological examinations were carried out separately for each block. Tissues showing characteristics of autolysis are excluded from our study.

### Immunohistochemistry

4µ sections cut from the Paraffin-embedded blocks were subjected to immunohistochemistry (IHC). Following deparaffinization at 70 °C to remove paraffin, antigen retrieval was carried out at 100 °C for 15 min in Tris EDTA buffer solution (pH 6.0). Polyexcel peroxidase quencher, a peroxide blocker, was incubated for 10 minutes to inhibit endogenous peroxidase activity. Each antibody was then incubated over the tissue section for 45 minutes, followed by incubation with a secondary antibody for 30 minutes. Mayer’s hematoxylin was used as a counterstain, and IHC staining was evaluated under the microscope. Tumor grading was performed based on the World Health Organization (WHO, 2019) and College of American Pathologists (CAP) criteria. Grading was determined by evaluating the degree of glandular differentiation, nuclear atypia, and mitotic activity on hematoxylin and eosin (H&E)-stained sections.

### Assessment of immunostaining

Staining intensity was evaluated using Image J. A threshold was set, and PD-L1 immunohistochemistry staining was analyzed by examining ten regions of interest (ROIs) from a single slide. Staining intensity (absent, weak, and strong) was evaluated. Specimens with < 5% tumor membrane staining were considered negative. The tumors were split into two groups for statistical analysis, with 17.519% expression used as the cutoff point based on the median expression of PD-L1. Those with less than the cutoff point expression were grouped as low PD-L1 expression, and those with high PD L1 expression were included in strong staining.

### Rna extraction and semiquantitative RT-PCR

The phenol-chloroform method was used to extract total RNA from the tissues. It was reverse-transcribed into cDNA. The following primer pairs and Qiazen SYBR-Green Master kit were used for PCR in compliance with the manufacturer’s instructions: GAPDH (forward, 5’- AGGAAGCTTGTCATCAATGGAAATC-3’; reverse, 5’- TGATGACCCTTTTGGCTCCC −3’); and PDL1(forward,5’-GCCGAAGTCATCTGGACAAGC-3’; reverse,5’- GTGTTGATTCTCAGTGTGCTGGTCA −3’).

### Western blotting

Sigma’s protease inhibitor cocktail (1: 100) and the RIPA buffer (20 mM Tris, pH 7.4, 150 mM NaCl, 1% Triton X-100, 1% Na deoxycholate, 2 mM EGTA, 2 mM EDTA, 0.1% SDS) were added to the phosphatase inhibitor cocktails (1:200; Sigma). The cells were then lysed in this solution following a PBS wash. Following a 10-minute centrifugation at 14,000 ×g at 4 °C, the lysates were subjected to the BCA protein quantification test to equalize the total protein concentrations. A SDS-PAGE gel was then loaded with 30 µg of total protein from each sample, and the primary antibodies (1:500, rabbit anti-PD L1 Thermofisher Scientific, CA; 1:1000, mouse anti-beta actin; Santa Cruz Biotechnology; 1:500) and the relevant secondary antibodies were blotted overnight at 4 °C.

### Immunofluorescence

PDAC paraffin blocks are cryosectioned with each section being 5 μm thick. Following acetone fixation, tissue slices were stained with primary and fluorescently labeled secondary antibodies, and DAPI was used as a counterstain. The Olympus BX-63 slide scanner was used to get immunofluorescence pictures of the mounted tumor tissue slices. ImageJ (https://imagej.net/) was used to analyze the images. Ten areas of interest (ROIs) were chosen at random from each tumor segment, and signals were quantified by pre-processing threshold values.

### Follow-up

Following the completion of all treatments, patients were monitored every three months for the first two years then every six months for the following years.

## Result

### Radiological intensity histogram and texture analysis

**Fig. 3:**
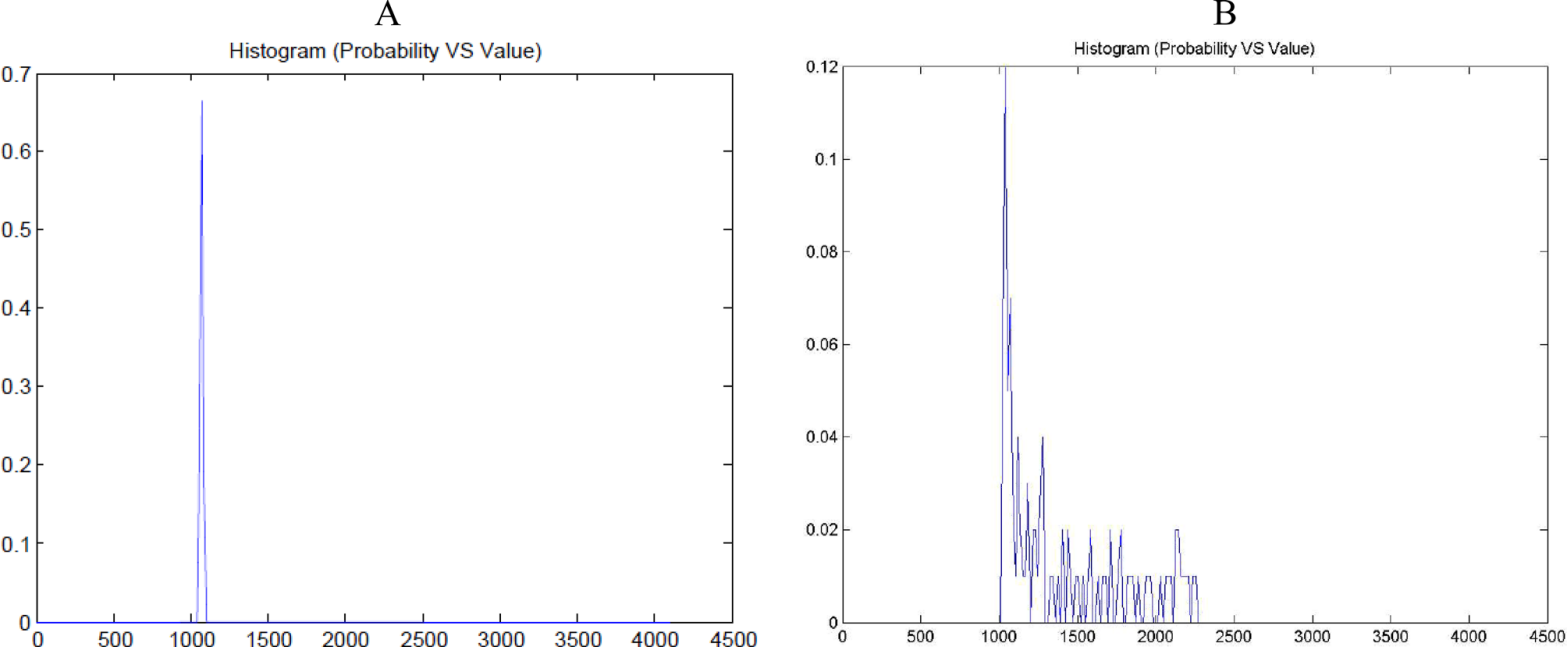
Intensity histogram (**A**) Normal pancreas, (**B**) PDAC

A comprehensive intensity histogram analysis generated from the ROIs highlights distinct patterns in pixel intensity distribution. The normal pancreas’s intensity histogram displayed a single, symmetrical peak (Fig. 3A). The histogram curve was narrow, with most pixel intensities clustered around a central value, indicating uniform contrast enhancement and tissue density.

**Fig. 4:**
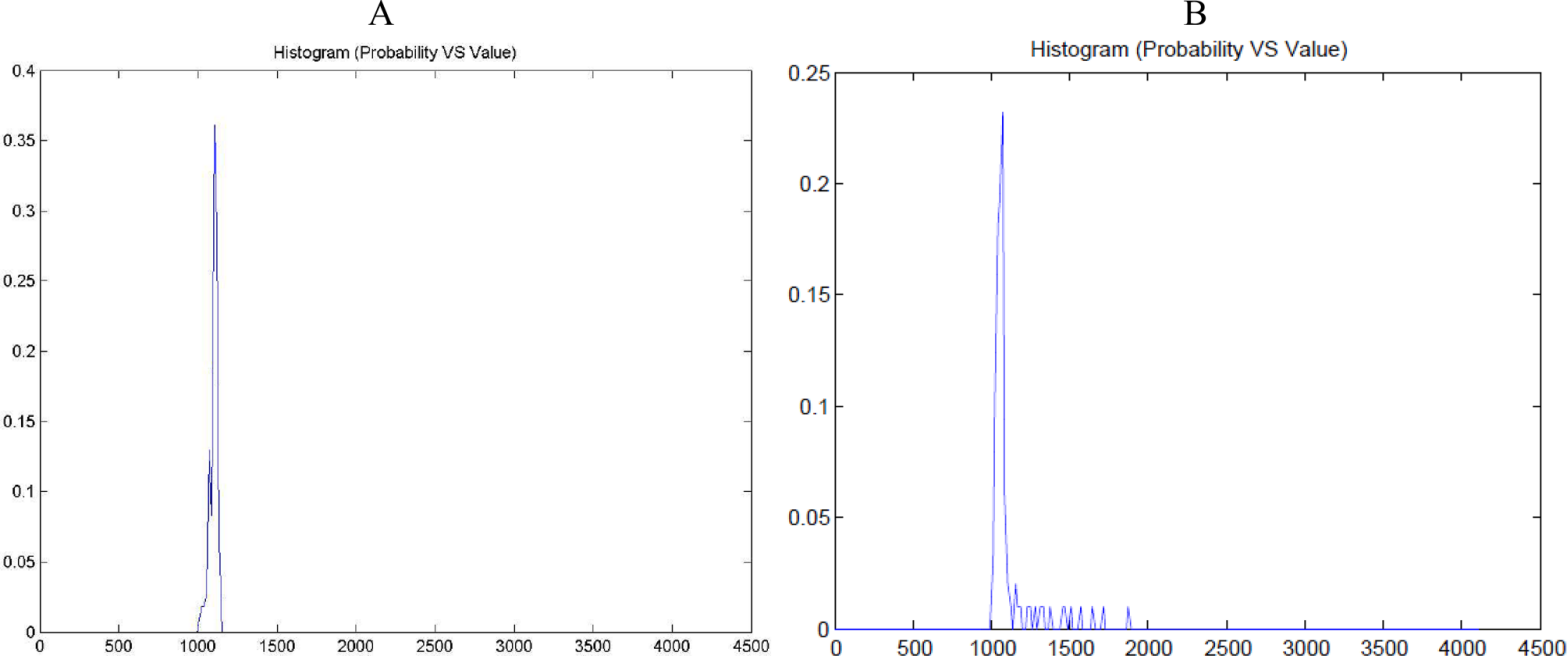
Intensity histogram peak height (**A**) lower PDL1 expression group, (**B**) Higher PD L1 expression group

Conversely, in PDAC tissues, histograms revealed multiple peaks and broader distributions, reflecting increased heterogeneity within the tumor microenvironment (Fig. 3B. These findings are consistent across all PDACs. Higher histogram height is correlated with lower PD-L1 expression; similarly, lower histogram height is related to higher PD-L1 expression (Fig. 4).

### GLCM texture analysis

**Fig. 5:**
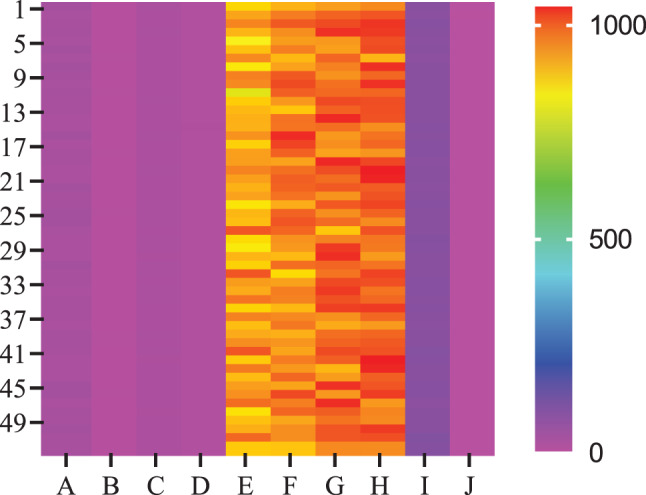
Heatmap represents radiomic texture parameters extracted from CECT scans of 52 pancreatic ductal adenocarcinoma (PDAC) patients. Each row corresponds to an individual patient (P1 to P52), while each column represents a specific GLCM-derived texture feature obtained using IBEX software. The features shown include A: Interquartile Range,B: Kurtosis,C: Mean Absolute Deviation,D: Median Absolute Deviation,E: 5th Percentile,F: 50th Percentile(Median),G: 75th Percentile,H: 95th Percentile, I: Range,and J: Skewness. The color intensity reflects scaled values of these parameters, with magenta indicating lower values and yellow to red indicating higher values, thereby illustrating the variation in texture heterogeneity and intensity across patients

**Fig. 6:**
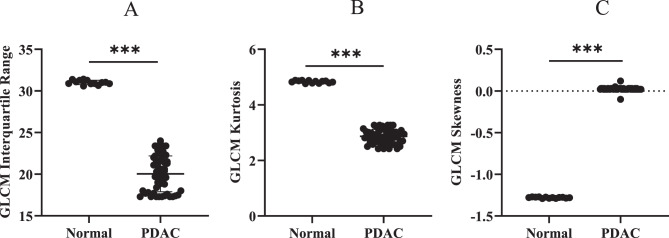
Quantitative radiomic analysis using the Gray Level Co-occurrence Matrix (GLCM) revealed significant differences in texture parameters between normal pancreatic tissue and pancreatic ductal adenocarcinoma (PDAC).(**A**) GLCM interquartile range (IQR) (**B**) GLCM kurtosis (**C**) GLCM skewness

In addition to histogram-based assessments, Gray Level Co-occurrence Matrix (GLCM) analysis was performed to evaluate spatial heterogeneity in PDAC regions. Interquartile Range, Kurtosis, Mean Absolute Deviation, Median Absolute Deviation, 5th Percentile, 50th Percentile, 75th Percentile, 95th Percentile, Range, and Skewness are extracted from CECT datasets for each patient (Fig. 5). Key texture features such as interquartile range, kurtosis, and skewness were compared between normal pancreas and PDAC and correlated with PD-L1 expression (Fig. 6).

Comparison between normal pancreatic tissue and pancreatic ductal adenocarcinoma (PDAC) regions revealed distinct patterns in select features. Specifically, Interquartile Range and Kurtosis were significantly higher in normal pancreas compared to PDAC (*p* < 0.05) (Fig. 6A, B), suggesting greater intensity variability in normal tissue. In contrast, skewness was significantly elevated in PDAC regions (*p* < 0.05), reflecting asymmetrical pixel intensity distributions likely due to tumor-induced heterogeneity (Fig. 6C).

**Fig. 7:**
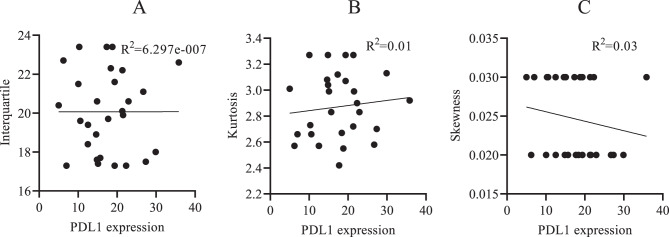
(**A**) Linear regression analysis between PD L1 expression and interquartile range. (**B**) Linear regression analysis between PD L1 expression and Kurtosis. (**C**) Linear regression analysis between PD L1 expression and Skewness

Subsequent correlation analysis between these radiomic features and PD-L1 expression showed no statistically significant associations (*p* > 0.05) (Fig. 7A-C). This indicates that although GLCM-derived texture metrics can differentiate between normal and malignant pancreatic tissues, they may not directly represent PD-L1 expression within the tumor microenvironment.

### PDL1 expression

**Fig. 8:**
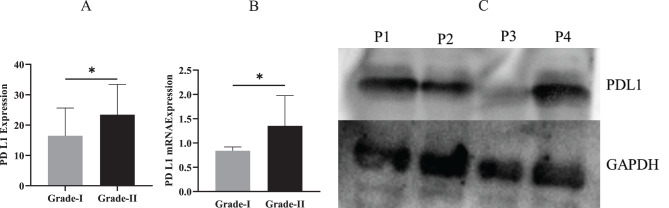
(**A**) Comparative analysis of PD L1 expression in Grade-I and Grade-II of PDAC. (**B**) Comparative analysis of PD L1 mRNA expression in Grade-I and Grade-II of PDAC. (**C**) Western blot analysis demonstrating inter-patient variability in PD-L1 protein expression

Programmed death-ligand 1 (PD-L1) expression was assessed by immunohistochemistry in a subset of 52 tumor samples. Among these, strong PD-L1 expression was observed in 28 cases (53.8%), while 24 cases (46.2%) showed no detectable PD-L1 expression. There is significant increase in PDL1 expression in Grade-II compared to Grade-I (Fig. 8A).

### RT-PCR analysis

PD-L1 mRNA expression was significantly upregulated in Grade-II PDAC tissues compared to Grade-I PDAC tissues. Specifically, the mean relative PD-L1 mRNA expression was 0.83 in Grade-I tumors and 1.35 in Grade-II tumors. This difference was statistically significant, indicating a higher transcriptional activity of the PD-L1 gene in more advanced pancreatic tumors (fold change: 1.60, *p* < 0.05) (Fig. 8B).

### Western blot analysis

Densitometric analysis of the normalized PD-L1 protein levels revealed a numerical trend of increased expression in Grade-II tumors as opposed to Grade-I. This observed difference, however, did not reach statistical significance (*p* > 0.05) (Fig. 8C). More research with a bigger sample size is necessary to validate this trend and its statistical significance.

### Immunofluorescence

**Fig. 9:**
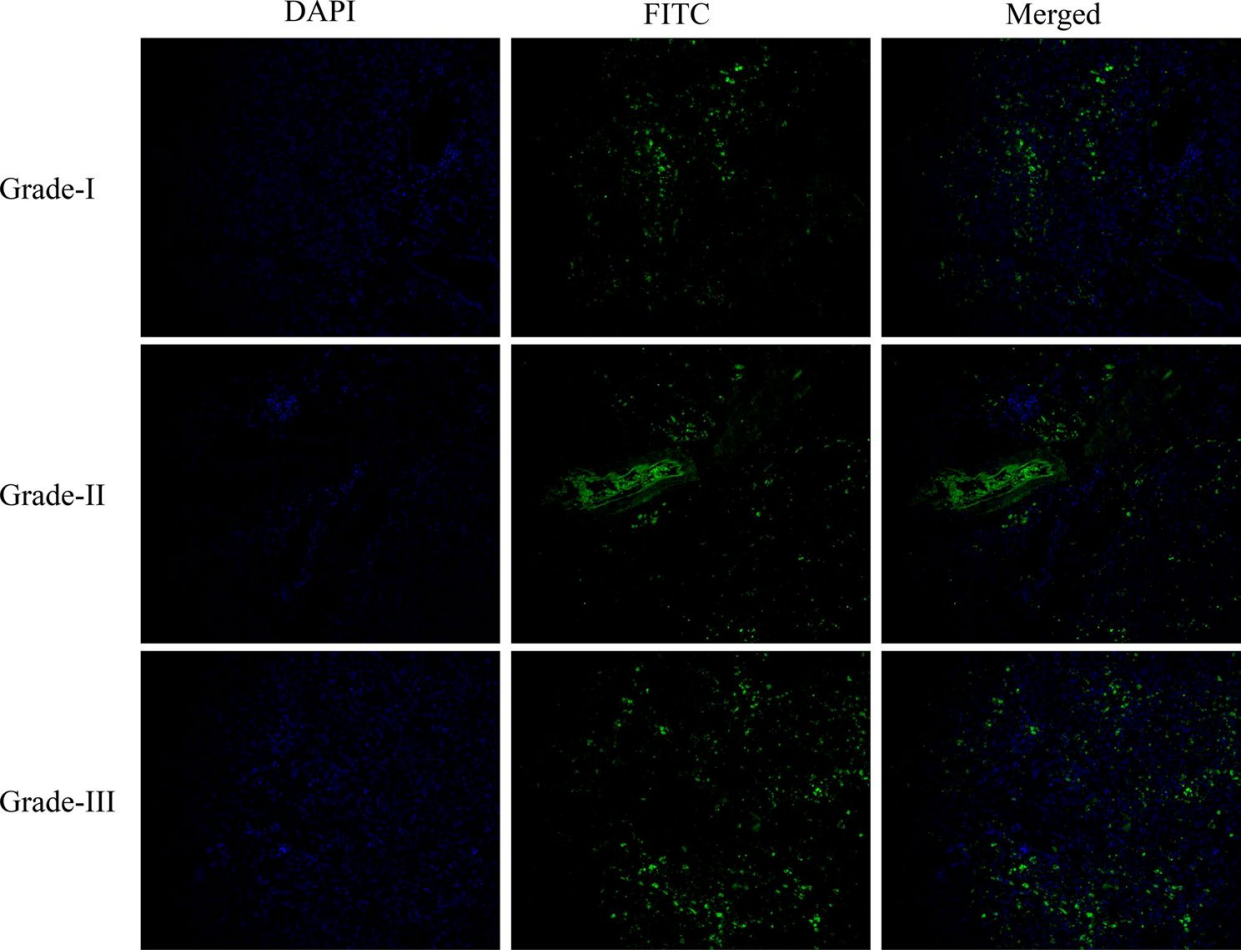
Immunofluorescence analysis of PD-L1 expression in different histological grades of PDAC (20X magnification)

A noticeable difference in PD-L1 expression was seen across tumor grades. In well-differentiated PDAC, PD-L1 staining was weak and mainly found in the glandular epithelial cells. On the other hand, poorly differentiated (Grade II) tumors had higher PD-L1 expression, showing a broader cytoplasmic distribution that extended into the stromal areas) (Fig. 9).

### Correlation between PDL1 expression and FDG uptake

**Fig. 10:**
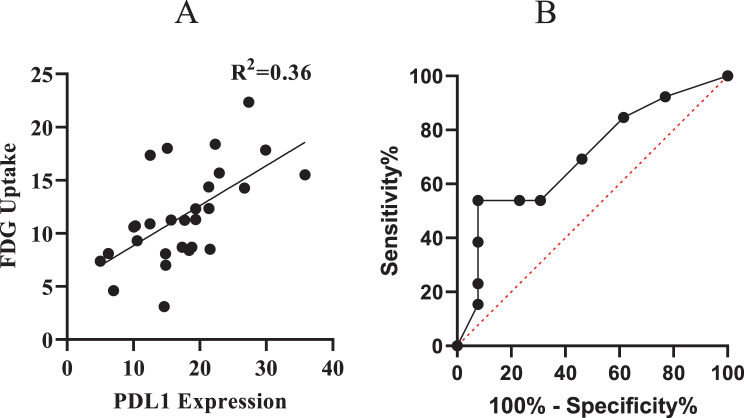
(**A**) Linear regression analysis between PD L1 expression and FDG SUVmax. (**B**) ROC curves evaluating the ability of FDG SUVmax to discriminate between high PD-L1 expression and low PD-L1 expression group in pancreatic cancer patients. The AUC for FDG: 0.7012 (95% ci: 0.4949–0.9074; *p* = 0.0812)

A statistically significant positive correlation was observed between PD-L1 expression levels and FDG uptake in PDAC patients. As shown in the scatter plot (Fig. 10 A), tumors with higher PD-L1 expression tended to exhibit high metabolic activity, as measured by SUVmax on PET/CT. The linear regression analysis yielded an R^2^ value of 0.36, suggesting a moderate association between PD-L1 expression and FDG uptake. ROC curve for FDG SUVmax showed an area under the curve (AUC) of 0.7012 (95% Confidence Interval: 0.4949–0.9074, *p* = 0.0812), indicating a fair discriminatory ability between high and low PD-L1 expression groups (Fig. 10B). The observed trend suggests that tumors with higher FDG uptake may be associated with increased PD-L1 expression.

### Correlation between PDL1 expression and FAPI uptake

**Fig. 11:**
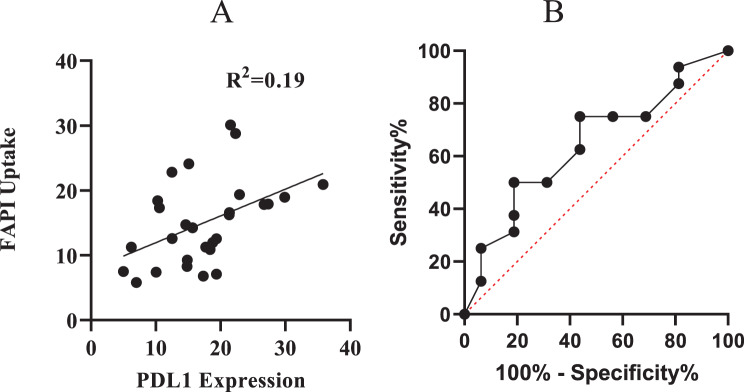
(**A**) Linear regression analysis between PD L1 expression and FAPI SUVmax. (**B**) ROC curves evaluating the ability of FAPI SUVmax to discriminate between high PD-L1 expression and low PD L1 expression group in pancreatic cancer patients. The AUC for FAPI: 0.6 (95% ci: 0.4–0.8; *p* = 0.1689)

Linear regression analysis between PD-L1 expression levels and FAPI uptake demonstrated a positive but weaker correlation compared to FDG uptake. The analysis yielded an R^2^ value of 0.19, suggesting that tumors with higher PD-L1 expression tended to exhibit slightly high FAPI uptake, indicating a potential interplay between immune checkpoint activity and stromal remodeling (Fig. 11A). FAPI SUVmax demonstrated an AUC of 0.6 (95% CI: 0.4–0.8, *p* = 0.1689), reflecting a lower discriminatory performance for PD-L1 status (Fig. 11B). The result indicates the limited utility of FAPI PET alone in predicting immune checkpoint expression. This finding supports the hypothesis that the desmoplastic tumor stroma, captured by FAPI PET, may contribute to immune evasion mechanisms through PD-L1 expression, albeit not as prominently as metabolic activity captured by FDG PET.

### Association between patients’ characteristics and PD-L1 expression

Age, tumor grade, CEA, and TNM stage did not significantly differ between the PD-L1-positive and PD-L1-negative groups (*p* > 0.05). In contrast, there was a substantial and statistically significant difference in both FDG SUVmax (*p* = 0.0223) and FAPI SUVmax (*p* < 0.0001) between the two groups.

### Kaplan–Meier survival analysis

**Fig. 12:**
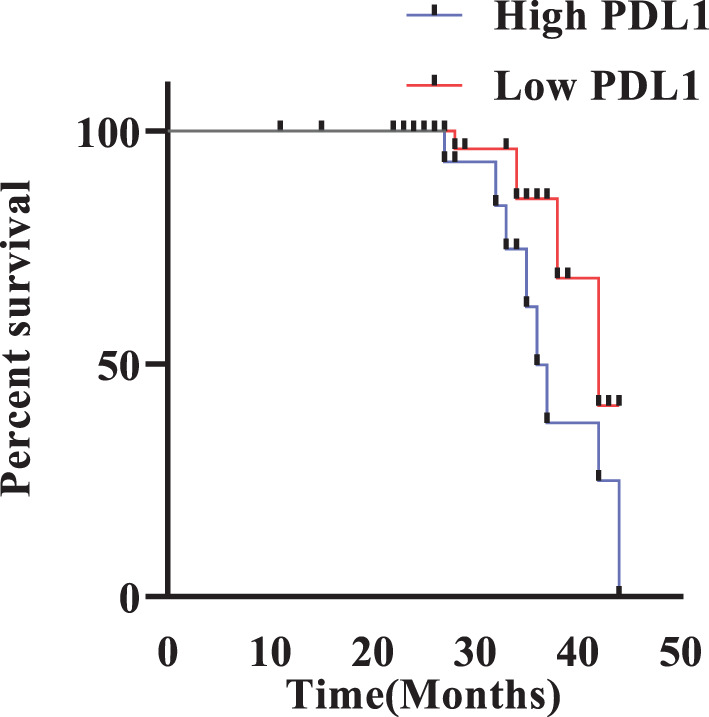
Survival analyses of PDAC patients with high and low PD-L1 expression groups

Kaplan–Meier survival analysis revealed that patients with high PD-L1 expression had significantly worse overall survival than those with low PD-L1 expression (Log-rank test, χ^2^ = 4.01, *p* = 0.0452). The median survival was 36.0 months in the high PD-L1 group and 42.0 months in the low PD-L1 group. The hazard ratio (HR) for death in high PD-L1 vs low PD-L1 was 3.24 (95% CI: 1.025–10.25), indicating a significantly increased risk of mortality associated with high PD-L1 expression (Fig. 12).

## Discussion

The spatial distribution of PD-L1 expression within tumors is increasingly recognized as a key determinant of immunotherapy efficacy [21]. PD-L1 heterogeneity significantly impacts the response to immune checkpoint inhibitors (ICIs), particularly those targeting the PD-1/PD-L1 axis. However, tumor regions with low or absent PD-L1 expression may reflect immune-excluded or immune-desert phenotypes [22–24], where insufficient T-cell infiltration or stromal barriers prevent effective immune activation. This intra-tumoral discordance can lead to partial or non-uniform therapeutic responses, with some tumor areas regressing while others progress, contributing to immune escape and resistance.

We evaluated the capacity of multimodal imaging CECT, MRI, and PET-CT to non-invasively assess PDL1 expression in PDAC. Our results underscore the reliability of these imaging platforms in capturing spatial variations in tumor architecture, metabolic behavior, and stromal composition, which are crucial determinants of immunotherapy response. CECT, with its high spatial resolution and contrast enhancement capability, effectively delineated heterogeneous enhancement patterns within PDAC lesions [25–27]. These patterns corresponded with variations in metabolic activity and stromal density, as further supported by changes in immunohistochemical findings. Imaging-based approaches that infer PD-L1 distribution through correlates such as texture features, metabolic heterogeneity, and radiomic signatures offer a promising avenue for non-invasive, whole-tumor assessment.

PET-CT, specifically using 18F-FDG and FAPI tracers, provided vital functional insights. FDG uptake highlighted regions of heightened metabolic activity, while FAPI PET-CT illuminated the cancer-associated fibroblast (CAF)-rich stroma, a major component of the desmoplastic reaction in PDAC. A positive correlation between FDG uptake and PDL1 expression shows PET/CT could be used to predict PD-L1 expression of PDAC [28]. PD-L1 regulates the expression of GLUT1 and HK2, the key enzymes of glycolysis, by activating the JAK-STAT pathway, thereby promoting glycolysis of PDAC cells and ultimately leading to the reorganisation of pancreatic ductal adenocarcinoma glucose metabolism [28–30].

Similarly, it was observed that PD-L1 expression and FAPI uptake were positively correlated. When compared to CAF-poor tumor models, anti-PD-L1 antibodies significantly slowed the growth of tumors with a high number of PD-L1-expressing CAFs [18, 20]. CAFs promote tumor immune escape by secreting certain cytokines and vesicles that cause cancer cells to produce PD-L1 [18, 20]. CAFs influence immunological efficacy by controlling immune cell differentiation and extracellular matrix remodeling, in addition to controlling the expression of PD-L1 on the surface of cancer cells [31]. In various solid tumors, CAFs impact PD-1/PD-L1 inhibitor immunotherapy [32]. Several important molecules, including TGF-β, Ln-γ2, Wnt2, and exosome molecules, have been identified based on the immunosuppressive effects and associated mechanisms of CAFs in resisting PD-1/PD-L1 immunotherapy. According to experimental data, stromal cells that express FAP interact with T cells at the tumor’s periphery and restrict effector T cell growth and intratumoral recruitment in a variety of malignancies. Prior research has demonstrated a high correlation between the distribution and uptake of Ga-68 FAPI −04 on PET and FAP expression, which has been verified by IHC in tumor tissues [33]. FAP is an antigen target that allows imaging probes to visualize its expression in vivo. Recent research has established the predictive effectiveness of [68 Ga] FAPI PET/CT as a non-invasive method to track the response of immunotherapy [34, 35]. Thus, in the age of immunotherapy, the PD-L1 expression level in conjunction with FAPI PET/CT, a non-invasive stereoscopic dynamic functional molecular imaging technique, may offer a useful strategy for tracking the response to immunotherapy and identifying patients who stand to gain from it. The current investigation discovered FAPI PET/CT might complement metabolic imaging in assessing tumor immune microenvironment and predicting immunotherapy response.

Importantly, correlation analyses between imaging-derived features and PD-L1 expression revealed that tumors exhibiting elevated PD-L1 levels frequently overlapped with zones of pronounced metabolic or stromal heterogeneity as identified on PET-CT. There are various cases where higher SUVmax did not yield a statistically significant correlation with PD-L1 expression. This spatial concordance suggests that the expression of PD-L1 within PDAC is not uniformly distributed across the tumor mass but is instead shaped by the underlying heterogeneity of the tumor microenvironment. Such spatial variability in immune checkpoint expression has significant implications for immunotherapeutic strategies, as it may influence both the responsiveness to immune checkpoint blockade and the selection of targetable tumor regions. Intratumoral heterogeneity encompasses variability in cellular composition, metabolic activity, stromal density, hypoxic gradients, immune cell infiltration, and expression of immune checkpoint molecules such as PD-L1 [36–38]. This spatial and temporal variation directly influences the tumor-immune dynamics, often leading to differential therapeutic responses within the same tumor mass.

This multi-parametric approach is particularly valuable in PDAC, where biopsy sampling may not fully capture the complexity of the tumor microenvironment due to its heterogeneity and fibrosis. As imaging continues to evolve with the integration of radiomics and artificial intelligence, the capacity to map tumor biology at a spatial resolution, once achievable only through pathology is becoming a reality [39, 40]. Therefore, tumor heterogeneity not only contributes to primary and acquired resistance to immunotherapy but also complicates the predictive value of single-site biopsies or bulk tissue analysis [41]. Imaging-based assessment of PDL1 expression, as demonstrated in this study, offers a non-invasive and whole-tumor perspective that can potentially improve patient selection for immunotherapy treatment. Where standard diagnostic biopsies may fail to capture this complexity, leading to misclassification of patients’ eligibility for immunotherapy, these insights are essential for advancing precision immuno-oncology in PDAC and beyond.

Current strategies to improve immunotherapy efficacy in PDAC focus on overcoming the highly immunosuppressive tumor microenvironment. These include targeting immunosuppressive myeloid cells (e.g., through CD11b, CSF-1 R, CCR2/5, and CXCR1/2 inhibition), enhancing dendritic cell function (via CD40 agonists and dendritic cell vaccines), and disrupting B cell–macrophage interactions using BTK inhibitors [42–44]. Additionally, remodeling the tumor stroma, such as degrading hyaluronan, is being explored to improve immune cell infiltration and drug delivery. Our work will contribute to the ongoing progress in immunotherapy clinical trials by providing insights into invasive PDL1 expression, which may help stratify patients and refine therapeutic approaches in PDAC. Such strategies can enhance patient selection, guide targeted biopsy, and support the development of combination therapies aimed at overcoming regional resistance within heterogeneous tumors. Future studies should focus on validating these imaging biomarkers in larger, prospective cohorts and integrating them into clinical decision-making frameworks. Furthermore, combining imaging data with genomic and transcriptomic profiling could enhance our ability to personalize treatment and predict resistance mechanisms in PDAC.

## Conclusion

We investigated the capacity of multimodal imaging, specifically CECT, MRI, and PET/CT using 18F-FDG and Ga-68 FAPI −04 FAPI tracers, to non-invasively assess PD-L1 expression and its critical spatial heterogeneity in pancreatic ductal adenocarcinoma (PDAC). Our comprehensive analysis revealed that PD-L1 expression is indeed heterogeneous within PDAC tumors, with strong expression observed in over half (53.8%) of the patient cohort. Crucially, we established significant correlations between imaging features and PD-L1 status. Furthermore, a statistically significant positive correlation was identified between elevated 18F-FDG uptake on PET/CT and higher PD-L1 expression, underscoring the link between tumor metabolic activity and immune evasion. While a weaker correlation was observed with FAPI uptake, this still suggests an interplay between the desmoplastic stroma and PD-L1 expression. Importantly, patients with high PD-L1 expression demonstrated significantly worse overall survival, highlighting its prognostic value. By offering a more comprehensive understanding of tumor biology, our work can enhance patient selection for immunotherapy, guide targeted biopsy strategies, and facilitate the development of more effective combination therapies aimed at overcoming regional resistance. Future larger-scale, prospective studies are warranted to further validate these imaging biomarkers and integrate them into clinical decision-making frameworks for personalized immuno-oncology in PDAC.

## Limitations

The study took place at a single center with a small sample size, which may limit statistical power. We did not validate radiomic features in an external group and did not apply corrections for multiple comparisons because this analysis was exploratory. Future studies involving multiple centers with larger datasets and validation methods are needed to confirm these initial findings.

## Electronic supplementary material

Below is the link to the electronic supplementary material.

Supplementary Material 1

Supplementary Material 2

Supplementary Material 3

Supplementary Material 4

## Data Availability

All data generated or analysed during this study are included in this published article [and its supplementary information files].
