# Supplementary figures and images for: Radiomic features of CECT and SUVmax of dual-tracer PET/CT reveal PD-L1 spatial heterogeneity in PDAC
Source: Cancer Imaging. 2026 Feb 14;26:41. doi: 10.1186/s40644-025-00960-3 (PMC13011674; doi:10.1186/s40644-025-00960-3)

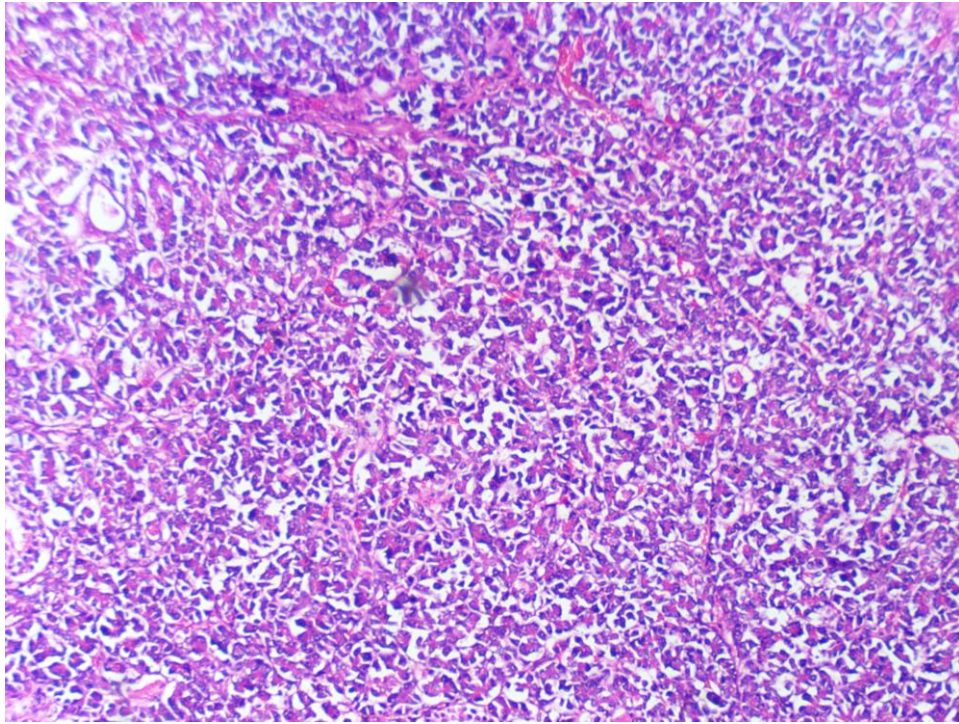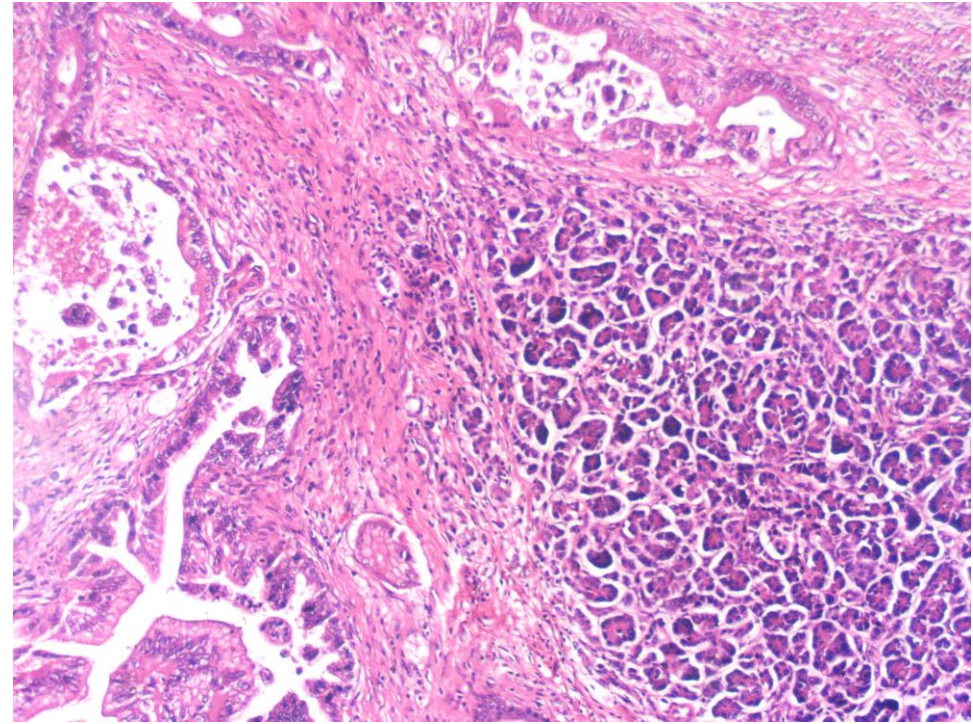

Supplementary file:Haematoxylin & Eosin (H&E) Staining to visualise autopsy samples

Supplement: Supplementary file 3 — Supplementary Material 3 [file 40644_2025_960_MOESM3_ESM.pdf]
